# Metastatic renal cell carcinoma initially presenting with hematochezia and subsequently with vaginal bleeding: a case report

**DOI:** 10.1186/s12894-018-0317-8

**Published:** 2018-01-30

**Authors:** Simon Ouellet, Audrey Binette, Alexander Nguyen, Perrine Garde-Granger, Robert Sabbagh

**Affiliations:** 1 Department of Surgery, Division of Urology, Université de Sherbrooke, Centre Hospitalier Universitaire de Sherbrooke (CHUS), 3001 12e avenue Nord, Sherbrooke, QC J1H 5N4 Canada; 2 Department of Obstetrics and Gynaecology, Université de Sherbrooke, Centre Hospitalier Universitaire de Sherbrooke (CHUS), 3001 12e avenue Nord, Sherbrooke, Canada; 3 Department of Pathology, Université de Sherbrooke, Centre Hospitalier Universitaire de Sherbrooke (CHUS), 3001 12e avenue Nord, Sherbrooke, Canada

**Keywords:** Renal cell carcinoma, Rectal metastasis, Vaginal metastasis

## Abstract

**Background:**

We report an unusual case of a synchronous rectal and metachronous vaginal metastatic renal cell carcinoma.

**Case presentation:**

A 78-year-old woman presented with hematochezia and a colonoscopy revealed a metastatic clear-cell renal cell carcinoma rectal polyp biopsy-proven. Abdominal computed tomography identified a 9.0-cm left renal mass with renal vein thrombosis, for which she underwent a laparoscopic radical nephrectomy. Histopathological examination confirmed a pT_3a_ clear-cell renal cell carcinoma. Seven months later, the patient presented with vaginal bleeding. Physical examination revealed a vaginal polypoid mass and biopsy confirmed a clear-cell renal cell carcinoma metastasis.

**Conclusions:**

This case represents unusual manifestations of metastatic renal cell carcinoma and is a reminder of the wide spectrum of clinical course of this disease.

## Background

Renal cell carcinoma (RCC) frequently metastasizes to the lungs, lymph nodes, bones and liver. Although RCCs have been shown to metastasize to virtually all organs, both rectal and vaginal metastases are exceptional. To our best knowledge, this is the fifth reported case of RCC metastasis to the rectum and the first one in a patient without a prior diagnosis of RCC [1–4]. Herein, we report a rare case of two unusual sites of metastatic clear-cell RCC (ccRCC) in a 78-year-old woman who initially presented with hematochezia and subsequently with vaginal bleeding due to synchronous rectal and metachronous vaginal metastases.

## Case presentation

**Fig. 1:**
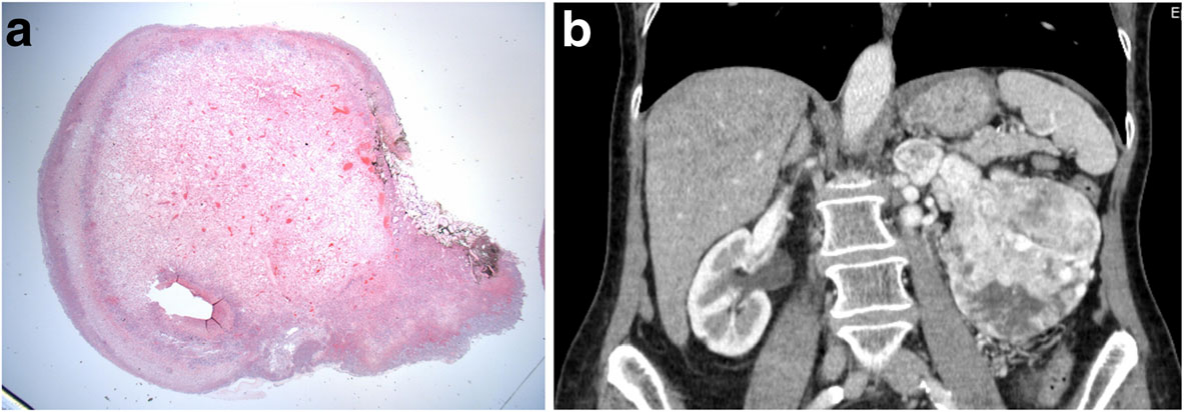
**a** Microscopic imaging (hematoxylin and eosin stain; 4×) of the tumoral proliferation with clear cytoplasm, infiltrating the rectal submucosal. **b** An abdominal computed tomography (CT) revealed a 9-cm heterogeneous and enhancing left renal mass with a 3-cm renal vein thrombosis

A 78-year-old woman consulted the gastroenterology outpatient clinic for painless hematochezia. She had no gross hematuria or abdominal pain. Her familial and past medical histories were unremarkable. Except for the presence of red stained stool on the digital rectal examination, the physical examination and laboratory investigations were within normal limits. A colonoscopy showed a 1-cm rectal polyp that was completely removed with a snare. Macroscopic examination of the specimen revealed an ulcerated rectal polypoid lesion with granulation tissue on its surface. Histologic examination exhibited proliferation of tumor cells disposed in layers and pseudo-glandular structures. The lesion was highly vascularized. The tumor cells had central nuclei, distinct borders and a clear cytoplasm suggesting clear cell carcinoma (Fig. 1a). Immunohistochemical studies of the tumor were positive for pankeratin, CD10, EMA and RCC-ma, confirming metastatic ccRCC. An abdominal computed tomography (CT) revealed a 9-cm heterogeneous and enhancing left renal mass with a 3-cm renal vein thrombosis (Fig. 1b). A chest CT was within normal limits.

**Fig. 2:**
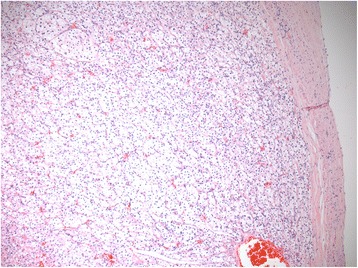
Microscopic imaging (hematoxylin and eosin stain; 100×) of the primary renal tumor

The patient then underwent a left radical laparoscopic nephrectomy, leading to the diagnosis of a clear-cell RCC (Fig. 2). The tumor measured 8 cm and was characterized as Fuhrman Grade III/IV and associated with a renal vein thrombus of 1.5 cm. The renal vein margin, the peri-renal fat and the left adrenal gland were not invaded and therefore the final pathological stage was pT3aR0. A pulmonary embolism complicated the postoperative period and the patient was started on anticoagulotherapy. The patient was considered cancer-free and no systemic adjuvant treatment was given. Chest and abdominal CT showed no signs of recurrence at 3 and 6 months.

**Fig. 3:**
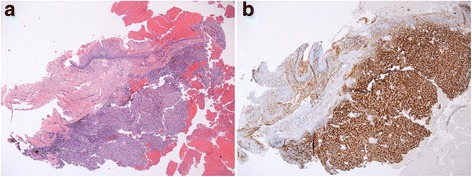
**a** Microscopic imaging (hematoxylin and eosin stain; 4×) showing islands of epithelial cells with clear cytoplasm nested in hemorrhagic fields under vaginal mucosa. **b** Immunohistochemical study demonstrating CD10 immunoreactivity. Tumour cells were negative for keratin 7

Seven months after the surgery, she presented with weakness, loss of appetite and sporadic vaginal spotting. Vaginal examination revealed a 3 and a 6-mm fragile polypoid lesion, both originating from anterior vaginal wall. There was no evidence of adenopathy or involvement of the vulva or cervix. Vaginal cytology was negative. A cystoscopy was performed to rule out a urethral diverticulum and was negative. A biopsy of both polyps was performed. Pathological analysis exhibited mucosal fragments containing foci of clear cell tumor, showing identical immunohistochemical staining features with primary tumor, such as diffuse staining with pankeratin, CD10 and RCC-ma (Fig. 3a and b). No systemic treatments were considered for the patient given her poor performance status.

Five months following the initial episode of vaginal bleeding, the patient was admitted to the hospital for persistent heavy bleeding. Physical examination revealed an important increase in size of the vaginal metastases. Laboratory tests were unremarkable, except for a hemoglobin level of 105 g/l. The patient received external beam radiotherapy with 20 Gray in 5 fractions directed to the vaginal lesions. Bleeding then diminished substantially. However, the patient expired 6 months later because of a rapid progression of her metastatic ccRCC (Table 1).

**Table 1:** Timeline

Time	Events
2007 – March	Painless rectal bleeding
2007- April	Colonoscopy and biopsy of a rectal polyp revealing metastatic ccRCC
2007 – May	Left radical laparoscopic nephrectomy – final pathology: ccRCC pT3a
2007 – December	Vaginal bleeding from a vaginal ccRCC metastasis
2008 – May	Significant vaginal bleeding from metastasis treated with external beam radiotherapy (20 Gray in 5 fractions)
2008 – October	Patient expired from disease progression

## Discussion and conclusions

Renal cell carcinoma (RCC) accounts for 3% of all adult malignancies and 85% of all primary renal tumors. RCC can present with a variety of symptoms due to local invasion, paraneoplasic syndrome and metastasis. Approximately 30% of patients with RCC present with metastatic disease at the time of diagnosis and 20 to 40% of those with initially localized disease will eventually develop metastasis [5]. RCC distant metastasis spread is lymphatic, haematogenous, transcoelomic or by direct invasion [6]. Frequent sites of metastasis from RCC include lungs, lymph nodes, bones and liver. Unusual sites of metastasis from RCC include thyroid, orbit, nasal structures, vagina, gallbladder, pancreas, sublingual tissues and soft tissues of distal extremities [6].

Gastrointestinal tract is rarely the site of metastatic lesions, although melanoma, ovarian and bladder cancer are most commonly involved [7]. Digestive RCC metastases are rare and tend to be present in patients with known RCC. The duodenum is the most commonly intestinal segment involved given its close location to the right kidney [8]. Upper gastrointestinal bleeding secondary to stomach and pancreatico-duodenal metastases have been described [5]. Only 13 cases of colon metastasis are reported and most of these patients presented with hematochezia [8, 9]. Rectal bleeding as the patient’s initial symptom of mRCC was seen in only one case [8]. Symptoms from bowel metastasis tend to occur more frequently in patients with know metastatic disease or in patients with a remote history of nephrectomy for RCC. Treatments for bowel metastasis include segmental resections, trans-catheter embolization, palliative derivation and endoscopic resection [5]. Endoscopic resection can be both diagnostic and therapeutic when the lesion is small as in this case.

Rectal metastasis from RCC is a very unusual event with only four cases in the literature (Table 2) [1–4].

**Table 2:** Clinical characteristics of previous cases of rectal metastasis of RCC

Author/Year	Sex/Age	Presenting symptom of rectal lesion	Prior nephrectomy	Timing after initial nephrectomy	Survival after initial GI bleeding
Current case	80/F	Hematochezia	No	–	18 months
Rosito et al., 2002 [2]	55/M	Anal bleeding	Yes	9 months	18 months
Dellon and Gangarosa, 2006 [1]	70/M	Hematochezia	Yes	28 years	11 months
Maehata et al., 2016 [3]	61/M	Hematochezia	Yes	NR	NR
Zheng et al., 2017 [4]	65/M	During follow-up for a benign rectal polyp	Yes	10 years	Alive with lungs, lymph nodes and bone metastasis

*NR* Not Reported

All patients had prior nephrectomy for RCC. Reported survival after initial symptoms is poor. Herein we present the first case of metastatic RCC initially presenting with hematochezia secondary to rectal metastasis.

Later in the course of the disease the patient presented with vaginal bleeding secondary to anterior vaginal wall metastases. Since primary adenocarcinoma of the vagina comprises less than 10% of all vaginal neoplasms, they should be considered metastatic until proven otherwise [10]. Metastatic adenocarcinoma of the vagina may develop from the cervix, endometrium, colon or ovary in 65% of cases [11]. Rarely, the primary tumor originates from the pancreas, the stomach, or exceptionally the kidney [10]. To date less than 100 cases of vaginal RCC metastasis have been reported in the literature [12]. In most of these cases, the vaginal lesion is typically solitary and located in the lower third of the anterior wall of the vagina [12]. Interestingly, the primary renal lesion is typically on the left side [11]. Retrograde venous dissemination seems the most plausible cause at the origin of vaginal metastasis, especially in our case with the presence of a renal vein thrombus. Immunohistochemically, metastatic clear cell carcinomas (CCC) to the gynecologic tract show constant positivity of CD10, which is in sharp contrast with the constant negativity of all primary gynecologic CCC, regardless of the site of origin. No conclusive data exist in the literature regarding the value of cervicovaginal cytology both in the diagnosis and the follow-up of these patients. Local excision and/or radiotherapy have been advocated as therapeutic interventions, although literature is limited [12].

In conclusion, we describe a rare case of synchronous metastatic RCC in a patient initially presenting with hematochezia secondary to metastatic involvement of the rectum. Subsequently the patient presented with vaginal bleeding secondary to metachronous vaginal metastases. This case illustrates the wide variability in RCC presentation and contributes to a better understanding of metastases to the rectum and the vagina.

## Abbreviations

CCC: Clear cell carcinoma
ccRCC: Clear-cell renal cell carcinoma
RCC: Renal cell carcinoma
